# Durable *HTT* silencing using non-evolved dCas9 epigenome editors in patient-derived cells

**DOI:** 10.1016/j.omtn.2025.102561

**Published:** 2025-05-14

**Authors:** Jennifer J. Waldo, Julian A.N.M. Halmai, Ankita Singh, Casiana E. Gonzalez, Yi-An Chen, Shaylyn A. Carthen, Jan A. Nolta, Kyle D. Fink

**Affiliations:** 1 Ctr. for Interventional Genetics, University of California Davis Health, Sacramento, CA, USA; 2 MIND Institute, University of California Davis Health, Sacramento, CA, USA; 3 Stem Cell Program, University of California Davis Health, Sacramento, CA, USA; 4 Gene Therapy Center, University of California Davis Health, Sacramento, CA, USA; 5 Institute for Regenerative Cures, University of California Davis Health, Sacramento, CA, USA; 6 Department of Neurology, University of California Davis Health, Sacramento, CA, USA

**Keywords:** MT: RNA/DNA Editing, CRISPR, epigenetics, dCas9, DNA methylation, Huntington’s disease

## Abstract

Huntington’s disease (HD) is an autosomal dominant neurodegenerative disorder caused by a trinucleotide repeat expansion in exon 1 of the huntingtin (*HTT*) gene. Nuclease-deficient Cas9 protein (dCas9) epigenetic editing for targeted gene regulation is a promising therapeutic approach for HD through downregulation of the causative gene, *HTT*. A screen of several dCas9 variants with expanded PAM recognition was fused to KRAB and DNMT3A/L to assess the ability to downregulate total *HTT*. Surprisingly, only *Sp*dCas9 could significantly downregulate *HTT*, while expanded PAM recognition variants dxCas9 and dCas9-VQR were less efficient or unable to reduce *HTT* expression. Using our lead construct with *Sp*dCas9, DNA methylation changes were assessed through reduced representation bisulfite sequencing, showing high on-target increases in DNA methylation and few off-targets. In addition, *HTT* silencing was mitotically stable for up to 6 weeks in a rapidly dividing cell line. Finally, significant downregulation of *HTT* was achieved in patient-derived neuronal stem cells, showing the efficacy of this system in a disease-relevant cell type. This approach represents a novel therapeutic pathway for the treatment of HD.

## Graphical abstract

**Figure:**
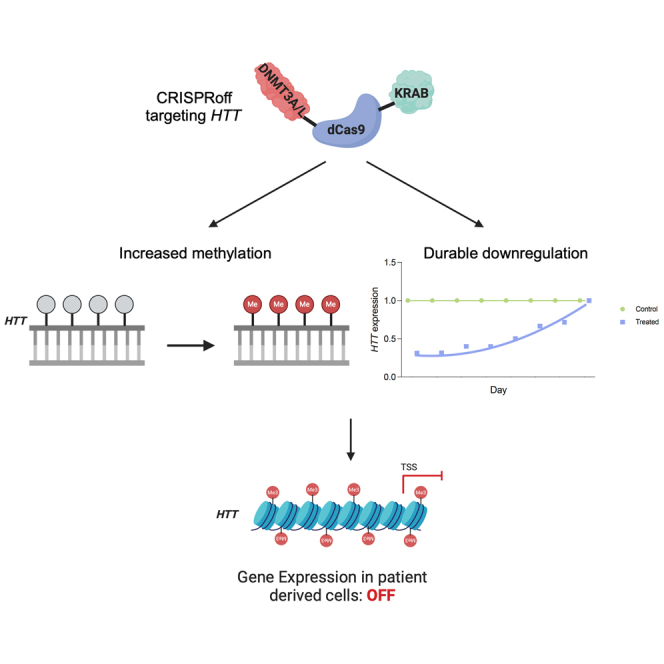

## Teaser

Targeting the huntingtin promoter using CRISPRoff results in significant silencing of HTT across multiple cell types including patient-derived neuronal stem cells. This effect is driven mechanistically through targeted promoter methylation and accumulation of repressive histone marks. The silencing of HTT was proven to be highly selective and durable.

## Introduction

Huntington’s disease (HD) is a fatal autosomal dominant neurodegenerative disorder characterized by progressive motor, cognitive, and behavioral symptoms. HD is fatal within 10–15 years of motor symptom onset, and there are currently no cures, with all treatments focusing on palliative care ineffective for modifying disease progression. HD is caused by a trinucleotide repeat expansion in exon 1 of the *huntingtin* (*HTT*) gene that causes widespread molecular dysfunction and cell death within neurons.^1^^,^^2^ The trinucleotide expansion leads to protein misfolding that generates a gain-of-function protein that has increased binding partners, creates protein aggregates, and causes neuronal intranuclear inclusions within neurons.^3^^,^^4^^,^^5^ Expression of mutant HTT affects many pathways within the cell, including energy metabolism, transcription, inflammation, and synaptic function.^6^^,^^7^^,^^8^^,^^9^^,^^10^ HD cellular dysfunction occurs on every level of the central dogma, from DNA slippage in replication, RNA toxicity, and protein misfolding and aggregation.^4^^,^^11^^,^^12^ This presents a unique problem as traditional therapeutics such as antisense oligonucleotides (ASOs) or antibodies may be less effective due to the toxicity of mutant *HTT* at the transcript level. The multifactorial dysfunction caused by mutant HTT warrants the investigation of DNA targeting with a CRISPR-based system.

While CRISPR-Cas9^13^ as a nuclease is a powerful tool, it is not necessarily applicable to disorders such as HD, as the repetitive nature of the causative gene makes precise excision challenging. There is also known pathogenesis of toxic N-terminal HTT fragments, making the induction of a premature stop codon an unlikely solution.^14^ Additionally, Cas9 nuclease induces permanent double-stranded breaks, which are known to cause unintended genomic alterations and inactivation of the p53 tumor suppressor pathways.^15^^,^^16^^,^^17^^,^^18^^,^^19^^,^^20^^,^^21^^,^^22^^,^^23^^,^^24^^,^^25^^,^^26^ This makes using a nuclease-deficient CRISPR system that could still affect gene expression an increasingly attractive prospect.

Using a nuclease-deficient Cas9 protein (dCas9) has allowed for the targeted regulation of gene expression via the fusion of effector domains for epigenetic editing.^27^ Effector domains or proteins involved in the reading, writing, and erasing of epigenetic marks can be fused to dCas9 to induce the modification of histone marks and DNA methylation. The KAP1/SETDB1 complex recognizes H3K9me3, which marks a heterochromatin state and signifies inactive genes.^28^^,^^29^ KRAB recruits the KAP1/SETDB1 complex to initiate the formation of heterochromatin and is the most potent and widely used effector domain for the downregulation of a target gene, with the effect generally only persisting for short periods.^28^^,^^29^ DNA methylation in the promoter region has also been highly associated with repressed genes.^30^^,^^31^ DNMT3A catalyzes *de novo* DNA methylation by recruiting its binding partner DNMT3L. The fusion of DNMT3L to DNMT3A increases the potency of the downregulation of gene expression over DNMT3A alone as it no longer relies on the recruitment of endogenous DNMT3L. Recently, Nuñez et al. showed the ability to downregulate gene expression for up to 15 months in rapidly dividing cells using a combination of KRAB (H3K9me3) and DNMT3A/L (DNA methylation) fused to a single dCas9 protein termed CRISPRoff.^32^ The combinatorial effect of the histone methylation and the mitotically stable DNA methylation not only improved downregulation but also promoted the stability of downregulation across generations of cells. Importantly, while this effect is persistent, it was also shown to be reversible through the removal of DNA methylation.^32^ This presents a unique opportunity to use epigenetic editing as a therapeutic approach for gain-of-function disorders such as HD, where the sustained downregulation of a single gene could have robust effects on disease phenotypes.

In this study, we used the CRISPRoff system to induce potent downregulation of *HTT* as a potential therapeutic for HD.^32^ We compared the ability of dCas9 variants VQR and dxCas9 to downregulate *HTT,* and found only *Sp*dCas9 was potent in its downregulation likely due to increased *HTT* promoter binding. We showed the ability to downregulate *HTT* in 3 different cell types: HEK293, K562, and patient-derived neuronal stem cells (NSCs). We performed a comprehensive assessment of DNA methylation showing that the downregulation was due to the large increase in methylation at the *HTT* promoter with very few off-target DNA methylation changes throughout the genome. We also showed that this downregulation was durable, persisting over 6 weeks in rapidly dividing cells following a transient transfection. Finally, we demonstrated significant downregulation of *HTT* in patient-derived NSCs, showing efficacy in a disease-relevant system.

## Results

### CRISPRoff can significantly downregulate *HTT* in HEK293s

**Figure 1:**
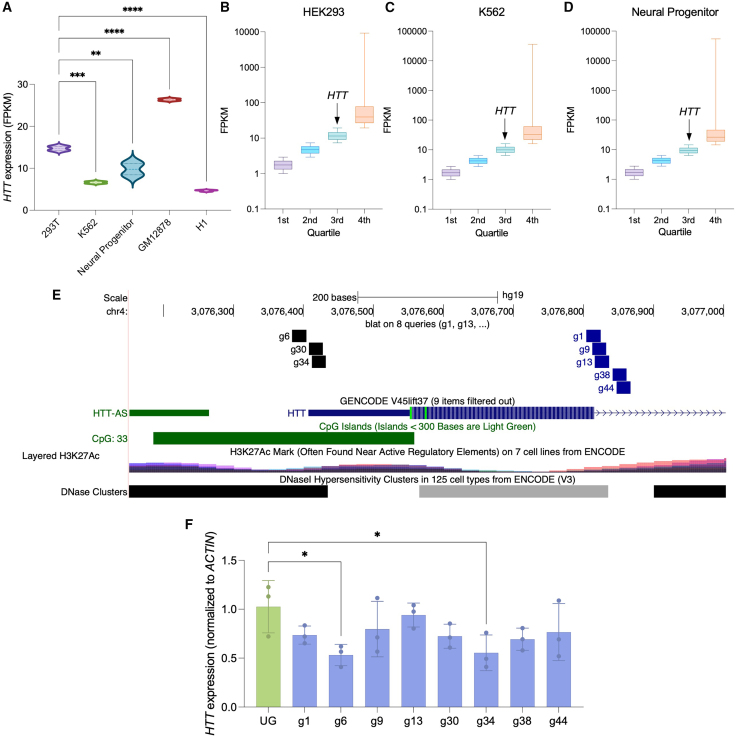
CRISPRoff can downregulate *HTT* in 293s (A) *HTT* expression in 5 cell lines based on fragments per kilobase of transcript per million mapped reads (FPKM). ∗∗*p* < 0.01, ∗∗∗*p* < 0.001, and ∗∗∗∗*p* < 0.0001 using a one-way ANOVA. (B–D) Quartiles of FPKM of all genes in (B) HEK293, (C) K562, and (D) neural progenitors. Only values above 1 FPKM were included. The box represents 25%–75% of values within that quartile, the line within the box represents the median value, and vertical lines represent the minimum and maximum values in that group. (E) UCSC hg37 genome browser track showing *HTT* TSS, gRNA binding sites, CpG island, H3K27ac, and DNAse hypersensitivity regions. (F) *HTT* expression of 293AAV cells transfected with the *Sp*dCas9 CRISPRoff system, taken down at 48 h post-transfection. ∗*p* < 0.05 using a one-way ANOVA, error bars represent SD.

To determine the effect of baseline *HTT* expression differences on CRISPRoff efficacy, HEK293 expression data were compared with *HTT* expression from other existing ENCODE cell line expression profiling data (Figure 1A). We found that expression of *HTT* in HEK293 cells (fragments per kilobase of transcript per million mapped reads [FPKM] = 14.73) was significantly higher than *HTT* expression in K562 (FPKM = 6.618), neural progenitors (FPKM = 9.772), and H1 induced pluripotent stem cells (iPSCs) (FPKM = 4.664) (Figure 1A). GM12878 lymphoblasts were the only cell type with higher HTT expression than HEK293s (FPKM = 26.34). Importantly, *HTT* expression was found moderately expressed and found within the 3^rd^ quartile of gene expression for HEK293 (Figure 1B), K562 (Figure 1C), and neural progenitors (Figure 1D), allowing for assessment of a downregulation approach in all 3 cell types. A total of eight sgRNAs were designed to target in or near the nucleosome-depleted transcriptional start site for efficient dCas9 binding.^33^ In addition, the *HTT* gene promoter contains a large CpG island that marks *HTT* amenable to epigenetic regulation via DNA methylation editing (Figure 1E). To identify *HTT* promoter-proximal regions amenable to CRISPRoff gene silencing, HEK293 cells were transiently transfected with a KRAB-dCas9-DNMT3A-3L construct and sgRNA expression vectors. CRISPRoff was co-transfected with an unguided control (UG) CRISPRoff (no sgRNA, UG) and assessed for *HTT* expression after 48 h in bulk samples. We find that sgRNA 6 could downregulate *HTT* by 47% (*p* = 0.0328), and sgRNA 34 was able to reduce *HTT* expression by 45% (*p* = 0.0441) (Figure 1F). Our findings are in accordance with genome-wide CRISPRi screens that highlight sgRNA position relative to the TSS dictates efficiency.

### Evolved dCas9 variants are inefficient at downregulating *HTT*

**Figure 2:**
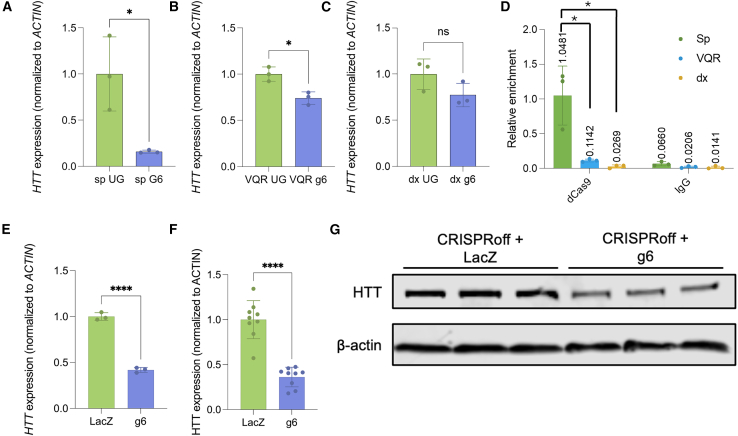
Evolved dCas9 variants do not induce robust downregulation of *HTT* in HEK293 cells (A–C) *HTT* expression was assessed 72 h after treatment with (A) *Sp*dCas9 CRISPRoff system, (B) VQR CRISPRoff system, and (C) dxCas9 CRISPRoff system. ∗*p* < 0.05 using a t test normalized to UG control. (D) ChIP-qPCR of dCas9 binding at *HTT* promoter, normalized to input. ∗*p* < 0.05 using a one-way ANOVA. (E) Paired qPCR analysis of *HTT* transcript levels in cells transfected with CRISPRoff and either sgRNA 6 or LacZ control. A fraction of the sample used for western blot analysis was allocated for RNA extraction. (F) Quantification of HTT protein levels by western blot across three replicates, normalized to β-actin. ∗∗∗∗*p* < 0.001 using a t test compared to LacZ control. (G) Representative western blot image of HTT in cells transfected with sgRNA 6 or LacZ control. Error bars represent SD.

In recent years, multiple Cas variants have been discovered or engineered to increase the targeting capabilities of Cas9 by expanding the PAM recognition capabilities of Cas9.^34^^,^^35^ Allele-specific targeting is considered critical for *HTT* therapy development but necessitates non-NGG PAM recognition to allow for SNP targeting. To elucidate the silencing capabilities of different engineered dCas9 CRISPRoff variants at the *HTT* locus, we then directly compared the ability of dCas9-VQR and dxCas9 to mediate *HTT* downregulation relative to *Sp*dCas9.^34^^,^^35^ Cells were transiently transfected with the respective CRISPRoff variant construct and sgRNA g6 or an UG, puromycin selected after 24 h, and total *HTT* expression was assessed 72 h post-transfection. *Sp*dCas9 showed significant downregulation of *HTT* using a single sgRNA with an 84% reduction (*p* = 0.0224) in *HTT* expression (Figure 2A). dCas9-VQR showed a moderate but significant downregulation of 26% (*p* = 0.0122) and dxCas9 showed no significant downregulation of *HTT* (Figures 2B and 2C). The amino acid changes required for engineering novel Cas protein often occur in an active site and could negatively affect the conformational changes necessary for binding, decreasing overall efficacy at certain loci, along with increasing PAM-interrogation time therefore decreasing on-target binding.^36^^,^^37^ To understand the mechanism of the differences in downregulation capabilities, we investigated the target occupancy of the three variants. Chromatin immunoprecipitation (ChIP)-qPCR for dCas9 binding was performed to assess the binding affinities of the dCas9 variants to the *HTT* promoter. *Sp*dCas9 had significantly increased enrichment in the *HTT* promoter compared to VQR and dxCas9, which had a 10-fold (*p* = 0.0082) and 50-fold (*p* = 0.0053) less enrichment in the *HTT* promoter, respectively (Figure 2D). Therefore, *Sp*dCas9-based CRISPRoff was used moving forward as it showed the highest on-target silencing and binding activity.

### CRISPRoff can significantly reduce HTT protein levels in HEK293s

Given that HTT trinucleotide expansion leads to protein misfolding and disrupts numerous cellular processes, we sought to determine whether CRISPRoff-mediated silencing also reduces HTT protein levels. To assess this, we performed western blot analysis on AAV-293 cells transfected with CRISPRoff and either HTT-targeting sgRNA 6 or a LacZ control. A portion of the sample was used for paired knockdown analysis by qPCR, which confirmed significant downregulation of HTT transcript levels (Figure 2E). Consistent with this, we observed a significant reduction in HTT protein levels following CRISPRoff treatment (Figures 2F and 2G). These findings demonstrate that CRISPRoff effectively reduces HTT expression at both the transcript and protein levels.

### CRISPRoff induces robust DNA methylation of the *HTT* promoter

**Figure 3:**
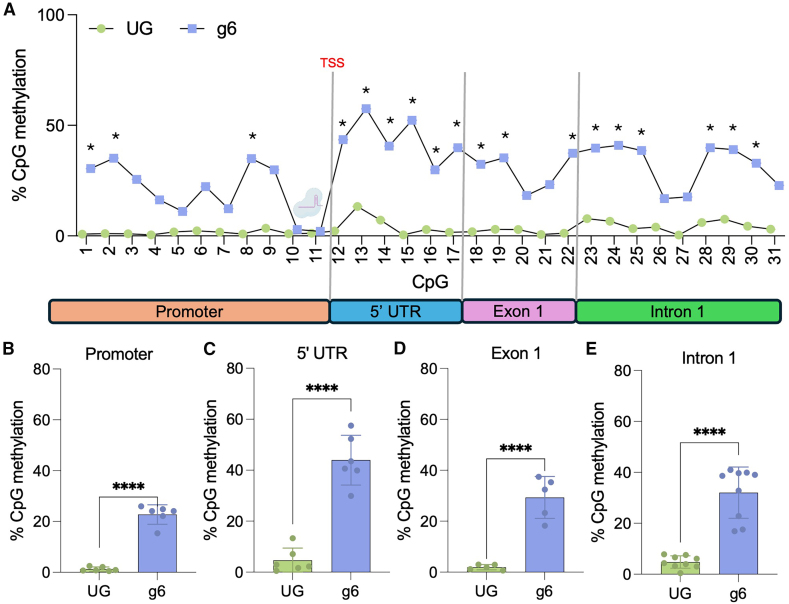
CRISPRoff induces robust methylation of *HTT* (A) Methylation of CpGs in HTT promoter and gene body. ∗ represents values with over 25% differential methylation status using a Fisher’s exact test, *p* value with a Benjamin Hochberg correction. Each CpG site had approximately 100 reads. Cas9-gRNA icon denotes the binding site of CRISPRoff with sgRNA 6. (B) Average methylation of all CpG sites in the *HTT* promoter. (C) Average methylation of all CpG sites in the *HTT* 5′ UTR. (D) Average methylation of all CpG sites in exon 1 of *HTT*. (E) Average methylation of all CpG sites in intron 1 of *HTT*. (B–E) ∗∗∗∗*p* < 0.001 using a t test compared to unguided control. UG, unguided. Error bars reoresent SD.

The methylation status of a gene is highly predictive of its transcriptional state, with highly methylated promoters corresponding to low transcriptional activity.^30^^,^^31^ Hypermethylation of certain gene promoters has also been shown to induce long-term downregulation, emphasizing the importance and functionality of DNA methylation editing.^38^ Reduced representation bisulfite sequencing (RRBS) was used to assess the methylation status of *HTT* in HEK293 cells to determine the mechanism of downregulation and target specificity. We observed a large coverage of CpG dinucleotides around our target site. In control-treated cells, little to no *HTT* CpG methylation was observed from promoter to intron 1 (3% ± 2.9% mean methylation) (Figure 3A). Following CRISPRoff treatment, DNA methylation was significantly increased across the promoter and into the gene body of *HTT* (Figure 3A). Interestingly, the only region without increased DNA methylation corresponded to the sgRNA binding site, which has previously been reported (Figure 3A).^39^ Cells treated with CRISPRoff had an approximately 22% increase (*p* < 0.0001) in DNA methylation across the promoter compared to the 1% DNA methylation of the control (Figure 3B). Significant increases in DNA methylation were also seen across the 5′ UTR (39% increase, *p* < 0.0001, Figure 3C), exon 1 (27% increase, *p* < 0.0001, Figure 3D), and intron 1 (27% increase, *p* < 0.0001, Figure 3E). Our findings show the ability of CRISPRoff to induce robust DNA methylation throughout the *HTT* promoter.

### CRISPRoff induces few off-target hypermethylated genes

**Figure 4:**
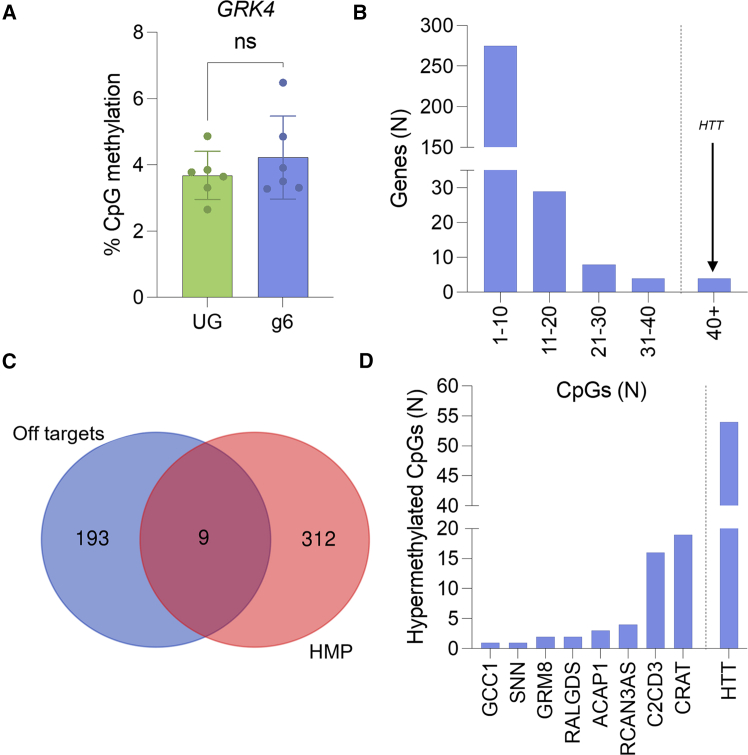
RRBS shows few off-target effects on DNA methylation in 293s (A) Average promoter methylation of nearest neighbor gene, *GRK4*. (B) Number of genes with different ranges of hypermethylated CpGs. Error bars represent SD. (C) Venn diagram of predicted *in silico* off-target sites based upon sgRNA 6 sequence with genes with hypermethylated promoters (HMP). *In silico* off targets included regions with up to 3 mismatches and 2 DNA/RNA bulges via CRISPR RGEN. (D) Number of differentially methylated CpGs of genes found in both in silico predicted off-targets and hypermethylated promoters in Figure 4C.

Off-target DNA methylation editing has been demonstrated for dCas9-DNMT3A fusion constructs, which could cause aberrant gene expression and unintended consequences.^40^ RRBS was used to enrich for CpG-rich regions and determine genome-wide off-target hypermethylation to understand the specificity of our system. After treatment with CRISPRoff and sgRNA 6, the increase in DNA methylation was localized to the *HTT* promoter, as the promoter of the nearest neighboring gene *GRK4* (TSS ∼111 kb upstream from sgRNA 6 binding site) did not have significant differences in DNA methylation when compared to an UG (Figure 4A). A total of 321 genes had at least 3 hypermethylated CpG sites in the promoter (25% change in methylation status, false discovery rate [FDR] <0.01) after treatment, with the majority having under 10 CpGs hypermethylated, compared to the over 40 seen at the *HTT* promoter (Figure 4B). When hypermethylated sites were overlayed with predicted *in silico* off-target sites, only 9 were present in both groups, with all but 2 having under 5 CpGs hypermethylated (Figures 4C and 4D). *C2CD3* and *CRAT* have over 10 CpG sites methylated and are predicted *in silico* off-targets, warranting further investigation of these sites for transcriptomic changes in a more disease-relevant cell type (Figure 4D).

### CRISPRoff can induce long-term downregulation of *HTT* over 6 weeks in K562 cells

**Figure 5:**
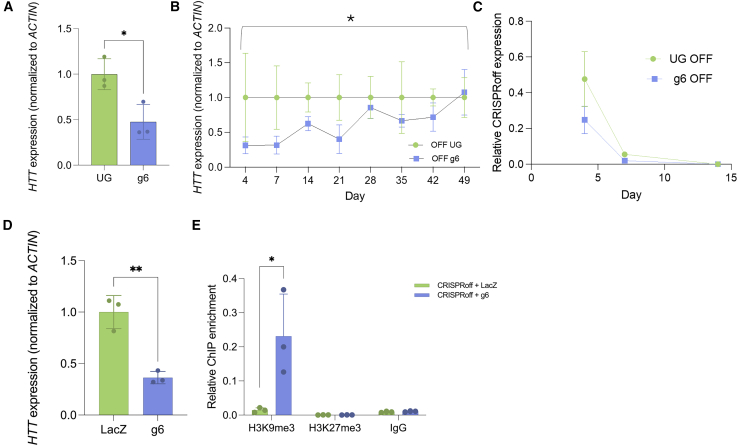
CRISPRoff can induce long-term knockdown of *HTT* over 6 weeks in K562 cells (A) *HTT* expression 4 days post-treatment. ∗ < 0.05 using a t test normalized to UG control. (B) Time course of *HTT* knockdown in K562 cells. Normalized to UG at each time point. ∗ < 0.05 using a three-way ANOVA across time. (C) Relative CRISPRoff expression over time, normalized to *ACTIN*. ∗ < 0.05 using a t test normalized to LacZ control. (D) Paired qPCR analysis of *HTT* transcript levels in cells transfected with CRISPRoff and either sgRNA 6 or LacZ control. A fraction of the sample used for ChIP-qPCR analysis was allocated for RNA extraction. ∗∗*p* < 0.01 using a t test compared to LacZ control. (E) Input normalized H3K9me3 enrichment levels determined by ChIP-qPCR of the HTT promoter. ∗*p* < 0.05 using a using a t test compared to LacZ control. Error bars represent SD.

The combination of DNMT3A/L and KRAB used in the CRISPRoff system has been shown to downregulate genes for weeks to months, warranting further investigation into the applicability of long-term downregulation at the *HTT* locus.^32^ K562 cells were nucleofected with CRISPRoff and sgRNA 6 or with an UG and puromycin selected after 24 h to select cells that had received the sgRNA plasmid. *HTT* expression was significantly downregulated at day 4, showing the efficacy of this system in multiple cell types (Figure 5A). *HTT* expression was then assessed over 7 weeks to determine the persistence of downregulation. *HTT* was significantly downregulated over 49 days when compared to an UG (Figure 5B). Notably, this downregulation was independent of the expression of CRISPRoff after 7 days, when dCas9 mRNA was no longer present within the treated cells (Figure 5C). These data highlight the ability to use CRISPRoff for hit-and-run epigenetic editing of a translationally relevant locus in a highly proliferative cell type.

### CRISPRoff induces enrichment of repressive H3K9me3 marks at the *HTT* promoter

As previously demonstrated, the KRAB domain recruits complexes that initiate heterochromatin formation through the deposition of H3K9me3. To assess whether CRISPRoff targeting of HTT results in similar epigenetic changes, we performed ChIP-qPCR in K562 cells nucleofected with CRISPRoff and either sgRNA 6 or a LacZ control. A fraction of the sample was allocated for paired knockdown analysis using qPCR, which confirmed significant downregulation of HTT expression in these cells (Figure 5D). ChIP-qPCR analysis revealed a significant increase in H3K9me3 enrichment at the HTT promoter (Figure 5E). In contrast, we observed no enrichment of H3K27me3 histone marks, supporting a specific mechanism of CRISPRoff-mediated silencing through H3K9 trimethylation (Figure 5E). These results demonstrate that CRISPRoff establishes a repressive chromatin environment at the HTT promoter, in part through the deposition of H3K9me3.

### CRISPRoff induces significant downregulation of *HTT* in patient-derived neuronal stem cells

**Figure 6:**
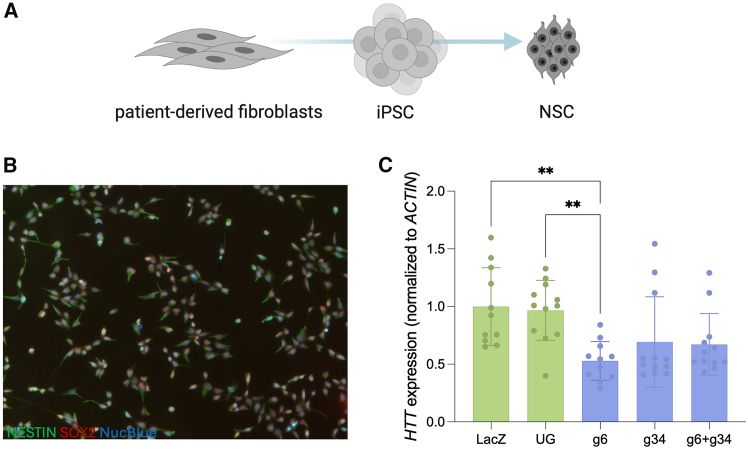
CRISPRoff induces knockdown of *HTT* expression in patient-derived NSCs (A) Schematic of differentiation of patient cells from fibroblasts to neuronal stem cells (NSCs). (B) Immunocytochemistry of ND36998C NSCs, nestin = green, Sox2 = red, NucBlue = blue. (C) *HTT* expression 48 h after nucleofection in ND36998G. Normalized to LacZ. ∗∗*p* < 0.01 using a one-way ANOVA, error bars represent SD.

To assess the efficacy of our epigenetic editing strategy on a more relevant cell line, patient-derived cells were treated with the CRISPRoff system. sgRNA screens were initially conducted in 4 HD patient fibroblast lines with a diverse range of CAG repeat lengths, but no effect was seen with any sgRNA, and expression of CRISPRoff was very low (ΔΔCT < 29) even after multiple rounds of optimization to increase transfection efficiency and cell health (data not shown). Patient-derived iPSCs were differentiated into NSCs to determine efficacy in a cell type that better recapitulates the primary cell type affected in the disease (Figure 6A).^41^ sgRNA 6 showed significant downregulation of *HTT* in the patient-derived NSCs when compared to an unguided and LacZ control (48% decrease, *p* < 0.05) (Figure 6C). However, the sensitive nature of the patient-derived NSCs did not allow for puromycin selection and represents a bulk effect, similar to bulk-transfected HEK293s (Figure 1F). There is a possibility that with improved delivery systems, the downregulation within patient cells could be increased. Interestingly, the multiplexing of sgRNA 6 and sgRNA 34 did not show synergistic downregulation of *HTT* (Figure 6C).

## Discussion

HD is a fatal disorder that currently only has palliative treatments and no cures. Epigenome editing provides a novel therapeutic approach via the downregulation of the causative *HTT* gene, decreasing expression of the mutant protein. In this study, we showed the ability to downregulate *HTT* in multiple cell types, including HEK293s, K562, and patient-derived NSCs. While *Sp*dCas9 showed robust downregulation, VQR and dxCas9 could not induce meaningful downregulation due to decreased *HTT* promoter binding. The CRISPRoff system induced significant methylation of the *HTT* promoter with few off-target hypermethylated genes, showing the specificity of this system. Finally, we achieved significant downregulation in a patient-derived NSC model, showing the application of this system in a disease-relevant model.

There is much debate within the HD field around the safety and efficacy of a non-allele-specific *HTT* reduction for HD. While *HTT* is known to have an important role in development, it is unclear what the consequences of a total loss of *HTT* would be in an adult human brain.^42^^,^^43^^,^^44^ While total *HTT* knockouts are embryonic lethal in mice, some groups have shown that knockouts of *HTT* in adult mice have limited to no effect.^44^^,^^45^^,^^46^ Despite this, there have been clinical trials for both total and allele-specific *HTT* reduction using ASOs and miRNAs, showing a clear clinical pipeline for both approaches.^47^^,^^48^^,^^49^ As over 99% of HD cases are caused by a single expanded allele, the prospect of allele-specific silencing is a promising approach.^50^ Interestingly, *HTT* resides in a conserved haplotype block with heterozygous SNPs that segregate with the expanded allele, allowing for allelic discrimination.^51^ Unfortunately, the SNPs in the *HTT* promoter are relatively devoid of NGG PAM sites recognized by *Sp*dCas9 used in the original CRISPRoff system. Therefore, the prospect of allele-specific downregulation necessitated the use of evolved dCas9 variants with differential or expanded PAM recognition. While many evolved Cas9 variants were developed to expand PAM recognition and increase binding efficiency, our data suggest that the evolved VQR and dxCas9 variants are far less efficient at downregulating *HTT*, likely due to their decreased binding efficiency to the *HTT* promoter. While it is known that the efficiency of downregulation via epigenetic editing is loci and effector domain-dependent, this study highlights the importance of assessing multiple dCas9 proteins, particularly when using evolved variants, to ensure optimal downregulation. Although our current system is targeting total *HTT*, it is possible that dCas proteins originating from another bacterial species or other evolved dCas9 variants would be capable of targeting disease-associated SNPs and downregulating *HTT* and should be investigated in the future.

Our approach showed significant increases in DNA methylation at the *HTT* promoter with few putative off-targets. Our system induced a 22% increase in methylation of the *HTT* promoter, with the increase continuing through exon 1 of the *HTT* gene. This effect seems to be localized to the *HTT* promoter, as it does not spread to the nearest neighboring gene *GRK4*, showing the ability to induce precise DNA methylation at our target. Although it was seen that 312 genes had hypermethylated promoters following CRISPRoff transfection, it is important to note that this effect may be due to the downregulation of *HTT* itself, as it is known to affect gene regulation.^52^ This made it imperative to compare predicted off-targets with the hypermethylated promoters to determine if the effect was likely due to CRISPRoff binding. The low number of hypermethylated promoters that are predicted off-targets show that the binding of CRISPRoff is relatively unpromiscuous and that the changes in the methylation status of most genes are likely due to the changes in *HTT* expression itself. The low number of CpGs methylated in most of the putative off-targets likely would not affect the expression of these genes, as the model of DNA methylation-based gene silencing proposes a high density of CpGs need to be methylated for stable silencing.^53^ Although unlikely, it is possible that the methylation of a single CpG can affect gene expression if it is in the binding site of a methylation-sensitive transcription factor-binding site.^54^ To fully elucidate the specificity of our system, ChIP sequencing should be completed to determine off-target binding *in vitro*, as well as RNA sequencing (RNA-seq) to determine if the levels of DNA methylation of the predicted off-target change gene expression in a meaningful way.

Our epigenetic editing approach for therapeutic development in HD has many advantages over traditional downregulation strategies such as ASOs and RNAi. The expanded *HTT* transcript can induce RNA toxicity, which cannot be fully addressed through RNA or protein-targeting therapeutics.^55^^,^^56^^,^^57^^,^^58^ Epigenetic editing also has the possibility of long-term downregulation, with multiple groups showing the ability to downregulate certain loci from 21 days up to 15 months in rapidly dividing cells.^32^^,^^59^ We were able to recapitulate this work, showing the sustained downregulation of *HTT* over 42 days in rapidly dividing cells. It is important to note that the previous studies showing long-term downregulation were of cell surface markers that allowed for the selection of only downregulated cells, likely increasing the efficacy and durability of the downregulation.^32^ This was not possible for *HTT* as it is not expressed on the cell surface, raising the possibility that the reactivation of *HTT* after 42 days could be due to a competitive advantage of the cells that did not have *HTT* downregulation. Additionally, the downregulation would likely be sustained for an even longer period in a post-mitotic neuron and could be an improvement over many ASOs that required dosing every 8–16 weeks and showed little improvement in disease phenotypes.^48^ It would be important for future studies to understand the kinetics of *HTT* downregulation in post-mitotic neurons to fully elucidate the longevity of this epigenetic downregulation in a more relevant cell type.

Finally, it is important to look at downstream phenotypes of HD to determine molecular rescue. Cells with expanded CAG repeats have well-characterized transcriptomic and morphological changes, as well as functional changes in energy metabolism and susceptibility to BDNF withdrawal.^60^^,^^61^^,^^62^ These phenotypes can be used as a proxy for phenotypic rescue and help determine if epigenetic downregulation of *HTT* using the dCas9 system is a robust candidate for the treatment of HD.

In conclusion, we induced robust downregulation of *HTT* using CRISPRoff. We showed that several evolved dCas9 editors were unable to meaningfully change *HTT* expression due to their decreased binding efficiency at the *HTT* promoter. The CRISPRoff system deposited significant DNA methylation at *HTT* with few off-targets and was able to downregulate *HTT* over 6 weeks. Finally, significant downregulation was seen in patient-derived NSCs, showing the efficacy of this system in a disease-relevant model. This study shows a future path toward epigenetic editors as a treatment for HD and other dominant neurological disorders.

## Materials and methods

### Gene expression analysis across multiple cell lines

RNA-seq counts for HEK293T (GSE146991),^63^ K562 (GSE175163),^64^ neural progenitor (GSE78635),^65^^,^^66^ GM12878 (GSE175228),^64^ and H1 (GSE187560)^64^ were all collected from their respective GEO repositories and the ENCODE project (https://www.encodeproject.org/) as FPKM. Genes were filtered for only those with expression over 1 FPKM.

### Plasmid generation

CRISPRoff-v2.1 was a gift from Luke Gilbert (Addgene plasmid no. 167981). To create the CRISPRoff-dxCas9 plasmid, the CRISPRoff backbone and gBlock containing dxCas9 were digested with SphI-HF and MluI-HF and ligated using T4 DNA ligase. The CRISPRoff-VQR plasmid was generated by digesting the CRISPRoff backbone and gBlock containing VQR with EcoRV and NotI and ligated using T4 DNA ligase. sgRNA 6 and 34 sequences were previously described.^67^ VQR sgRNA sequences were generated using the RGEN Cas-Designer tool (http://www.rgenome.net/cas-designer/).^68^^,^^69^ All sgRNAs were ordered as oligos from IDT. sgRNAs were cloned into an expression vector (plasmid no. 52963) according to previously established protocols.^70^ All constructs were sequence verified by Sanger sequencing (Genewiz, Inc.).

### Neuronal stem cell differentiation

iPSCs were differentiated into NSCs per previously published protocols.^41^ In brief, ND36998G iPSCs (Coriell, USA) were cultured on Matrigel (Corning)-coated plates and in StemFlex (Gibco) media. Cells were passaged every 3–4 days for maintenance. On day 1, 3 × 10^6^ cells/well were plated in 1 well of a 24-well Aggrewell 800 (StemCell Technologies) plate in embryoid body (EB) medium (KnockOut DMEM/F12 [Gibco] and 15% KSR [Gibco] plus 100× NEAA, 100× Glutamax, and 0.55 mM 2-mercaptoethanol). On day 2 cells were moved to Ultra-Low Attachment dishes (Corning) and media was changed to EB medium with 500 ng/mL Noggin (R&D Systems) and 10 μM SB431542 (Tocris). On day 4, media was changed with the EB media Noggin and SB431542. On day 5, EBs are plated onto growth factor reduced Matrigel with EB medium plus Noggin and SB431542. On day 6, media is changed to neural progenitor medium (Neurobasal Medium [Gibco] plus GlutaMAX, NEAA, 1% Pen/Strep, 1% N-2, and 20 ng/mL bFGF) and is changed every other day until day 12. On day 12, neural rosettes should be formed and passaged as single cells onto Poly-L-Ornithine/Laminin (Sigma) and Laminin (Sigma-Aldrich)-coated plates in NSC media (NPC media plus 0.1% B27, 20 ng/mL epidermal growth factor, 10 mg/mL insulin, and 1.6% D-glucose). NSCs were maintained in NSC media and passaged every 3–5 days on PLO/laminin-coated plates.

### Immunocytochemistry

ND36998G NSCs were seeded at 50,000 cells/cm^2^ in a 6-well plate. Twenty-four h after seeding, cells were fixed using 4% paraformaldehyde (PFA) for 15 min then subsequently washed with 20× Tris-buffered saline wash buffer for IHC/ICC (eBioscience). Cells were permeabilized using 0.5 mL permeabilization solution (4% PFA in PBS) for 15 min, followed by 0.5 mL 5% blocking solution (Fish Serum Blocking Buffer, Thermo Fisher Scientific) for 1 h at room temperature (RT). Primary antibodies Nestin (ab176571) and Sox2 (ab79351) were incubated for 3 h at RT. Wells were washed 3× times with wash buffer, followed by the addition of secondary antibodies, diluted in 500 μL of blocking solution and incubated at RT for 1 h. Wells were washed 3× with wash buffer, and 1mL of wash buffer was added with 2 drops/mL of NucBlue Fixed Cell Stain (Invitrogen) added 5 min before imaging. Cells were stored in the 4°C. Nikon Eclipse Ti-U inverted research microscope (Nikon, Melville, NY).

### Cell culture and transient transfections

All cells are cultured in incubators at 37 °C at 5% CO_2_.

AAV-293 cells (Agilent) were cultured in AAV-293 media composed of DMEM high glucose with 10% fetal bovine serum (FBS) and 1% L-glutamine. Cells were passaged every 5–7 days at approximately 80% confluency using Tryp-LE. AAV-293 cells were transfected using Lipofectamine 3000 (Invitrogen) according to the manufacturer’s protocols. In brief, 2.5 μg of the dCas9 constructs (CRISPRoff, VQR-off, or dxCas9-off) and sgRNA plasmid containing a puromycin selection marker in equimolar ratios, 5 μL p300, and 3 μL Lipofectamine 3000 were used per well in 500 μl of Opti-MEM (Gibco). Media was changed 24 h after transfection to AAV-293 media containing 1 μg/mL puromycin. Cells were lysed in TRIzol Reagent (Invitrogen) 72 h after transfection and stored at −80°C until processing.

K562s (ATCC) were cultured in RPMI with 10% FBS. K562 cells were electroporated using the Neon nucleofector (Thermo Fisher Scientific) 100 μl tips. One million cells/well were resuspended in T buffer with 10 μg total of CRISPRoff and sgRNA containing a puromycin selection marker in equimolar ratios. Conditions were set at 1,450 V, 10 mS, and 3 pulses. Cells were plated in a 6-well with 3 mL media per well. Media was changed after 24 h to K562 media containing 2 μg/mL puromycin. Media was changed to remove puromycin after 24 h. Cells were passaged every 3–5 days for maintenance. At each time point, 1 mL of culture media was removed and spun down at 1,500 rpm, and cells were lysed in 350 μl of TRIzol Reagent (Invitrogen) and stored at −80°C until processing.

NSCs were maintained in NSC media (Table S1) and passaged every 3–5 days on PLO/Laminin-coated plates. ND36998G NSCs were electroporated using the Neon Nucleofector (Thermo Fisher Scientific) 100-μl tips. Approximately ∼700k cells/well were resuspended in T buffer with 2 μg total of CRISPRoff and sgRNA in equimolar ratios. Conditions were set at 1,150 V, 20 mS, and 2 pulses. Media was changed after 24 h, and cells were lysed in 350 μl of TRIzol Reagent (Invitrogen) after 48 h and stored at −80°C until processing.

### Gene expression analysis using qPCRs

RNA was extracted using the Zymo RNA miniprep kits according to the manufacturer’s protocol. RNA (200–1,000 ng) was converted into cDNA using the iScript cDNA synthesis kit according to the manufacturer’s protocol. qPCRs were run on the QuantStudio Flex 6 (Applied Biosystems) with PowerTrack SYBR Green Master Mix (Applied Biosystems) using manufacturer settings. Experiments were done in biological and technical triplicates and were reported as fold changes following essential MIQE guidelines.^71^ All primers were designed using primer3 and are as follows: *HTT* (F: 5′ TGGATCTTCAGAACAGCACG 3′, R: 5′ TCGACTAAAGCAGGATTTCAGG 3′) and *ACTIN* (F: 5′ CTGGAACGGTGAAGGTGACA 3′, R: 5′ GGGAGAGGACTGGGCCATT 3′), and all calculations were done relative to *ACTIN*. For each sample, 12-μL qPCR reactions were set up as follows: 6 μL PowerTrack SYBR Green Master Mix (Applied Biosystems), 1.5 μL of primer (final concentration 0.5 μM for each primer), 3.5 μL H_2_O and 2 μL of cDNA sample (10 ng total). Thermal cycling conditions were as follows: 95°C for 1 s; 40 cycles of 95°C for 1 s, 60°C for 20 s; followed by a melt curve of 95°C for 15 s, 60°C for 1 min, and 95°C for 15 s.

### Western blot

Western blot was performed by harvesting AAV-293 cells 3 days post-transfection of CRISPRoff constructs and sgRNA plasmid. Cells were washed with PBS and protein lysates were harvested with Pierce RIPA Buffer (Thermo Fisher Scientific) supplemented with Halt Protease Inhibitor Cocktail (Thermo Fisher Scientific). Protein concentrations were quantified using the Pierce BCA Protein Assay Kit (Thermo Fisher Scientific). Protein lysate (20 μg) was mixed and heated with SDS loading buffer, separated on Bolt 4%–12% Bis-Tris Plus Gels (Thermo Fisher Scientific) and transferred onto a polyvinylidene fluoride membrane. Membranes were blocked with 10% Fish Serum Blocking Buffer (Thermo Fisher Scientific) in 0.1% TBST. Blots were then probed for HTT (MAB2166, Millipore Sigma) or actin (20536-1-AP, Proteintech) in 5% Fish Serum Blocking Buffer in 0.1% TBST overnight at 4 °C. Blots were washed with 0.1% TBST before incubation with secondary antibody (IRDye 680 Goat anti-mouse IgG [LI-COR, 1:5,000]) in 5% Fish Serum Blocking Buffer in 0.1% TBST for 1 h at RT. Blots were washed with 0.1% TBST and imaged on the Odyssey Clx (LI-COR). Bands were quantified with Empiria Studio.

### DNA methylation assessment using RRBS

DNA was extracted from cells using the DNA Miniprep Kit (QIAGEN, Hilden, Germany). Bisulfite conversion, library preparation, and sequencing were completed by the UC Davis Sequencing Core. In brief, Trim Galore was used to trim adapters, Bismark was used to align bisulfite-converted genomes to hg38, and SAMtools was used to sort the genome. Methylkit was used to analyze aligned reads. Low coverage CpGs (less than 10 reads covering bases) and exceptionally high coverage sites (coverage over 99.9% percentile) were filtered out. Differentially methylated bases were calculated compared to UG control. Bases were only considered hypermethylated if they had an over 25% increase in methylation compared to control. Output was annotated to hg38. Normalized read counts can be found in Table S1.

### ChIP and ChIP-qPCR

ChIP was performed according to previously published protocols.^59^^,^^72^ In brief, AAV-293 cells were seeded at 5 × 10^7^ in a 10-cm dish and transfected using Lipofectamine 3000 (Invitrogen) according to manufacturer’s protocols using 15 μg of the dCas9 construct and sgRNA plasmid containing a puromycin selection marker in equimolar ratios, 30 μL p300, and 18 μL Lipofectamine 3000 were used per well in 3 mL of Opti-MEM (Gibco). Media was changed to 293 media with 1 μg/mL puromycin after 24 h. Seventy-two h after transfection, cells were crosslinked using 1% formaldehyde at RT and the reaction was stopped with 0.125 M glycine. Cross-linked cells were lysed with ChIP lysis buffer (5 mM PIPES [pH 8] 85 mM KCl, and 1% Igepal) with a protease inhibitor (PI) cocktail (Roche). Nuclei were collected by centrifugation at 2,000 rpm for 5 min at 4°C and lysed in nuclei lysis buffer (50 mM Tris [pH 8], 10 mM EDTA, and 1% SDS) supplemented with PI cocktail. Chromatin was fragmented using the Bioruptor Pico (Diagenode, Denville, NJ, USA) and diluted with 5 volumes RIPA buffer (50 mM Tris [pH 7.6], 150 mM NaCl, 1 mM EDTA [pH8], 1% Igepal, and 0.25% deoxycholic acid). ChIP enrichment was performed by incubation with 5 μg hemagglutinin antibody (ab15069, Abcam) or 2 μg normal rabbit IgG (ab46540, Abcam) for 16 h at 4°C. Immune complexes were bound to 20 μL Pierce Protein A Magnetic Beads (Thermo Fisher Scientific) for 2 h at 4°C. Beads were washed 2 times with RIPA (Thermo Fisher Scientific) and 3 times with ChIP wash buffer (100 mM Tris [pH 8], 500 mM LiCl, and 1% deoxycholic acid). The final wash was performed in ChIP wash buffer with 150 mM NaCl. Cross-links were then reversed by heating beads in 100 μL ChIP elution buffer (50 mM NaHCO_3_ and 1% SDS) overnight at 65°C, and DNA was purified using the QIAquick PCR Purification Kit (QIAGEN). ChIP-qPCR targeting the *HTT* promoter was performed with PowerUp SYBR Green Master Mix (Applied Biosystems) using the QuantStudio 6 Flex (Applied Biosystems), and the QuantStudio software was used to extract raw CT values. Primer sequences were as follows: *HTT* F: 5′ GCCTCACCCCATTACAGTCT 3′ and *HTT* R: 5′ AGCATGATTGACAGCCCTAG 3′. ChIP enrichment was calculated relative to input samples using the delta CT method.

K562 cells were nucleofected with CRISPRoff and sgRNA 6 or a LacZ control and puromycin selected after 24 h to select cells that had received the sgRNA plasmid. Cells were cross-linked for 10 min in 1% formaldehyde 4 days after nucleofection. Cross-linking was stopped with 0.125 M glycine, washed in DPBS and cell pellets were stored at −80°C. ChIP analysis was performed using ChIP-IT Express Enzymatic kit (Active Motif). Briefly, chromatin was fragmented using the DNA shearing enzyme by incubation at 37°C for 10 min and shaken every 2 min. ChIP enrichment was performed by incubation with 2 μg H3K9me3 antibody (ab8898), 2 μg H3K27me3 antibody (ab6002), or 2 μg normal rabbit IgG (ab46540) in protein G magnetic beads. ChIP samples were rotated end-over-end overnight at 4°C. Samples were washed, eluted, and cross-links were reversed by incubation overnight at 65°C. DNA was purified using the QIAquick PCR Purification Kit (QIAGEN). ChIP-qPCR targeting the HTT promoter was performed with PowerUp SYBR Green Master Mix (Applied Biosystems) using the QuantStudio 6 Flex (Applied Biosystems) and the QuantStudio software was used to extract raw CT values. Primer sequences were as follows: HTT F: 5′ GCCTCACCCCATTACAGTCT 3′ and HTT R: 5′ AGCATGATTGACAGCCCTAG 3′. ChIP enrichment was calculated relative to input samples using the delta CT method.

### *In silico* off-target analysis

Off-target analysis of CRISPR sgRNA was done using the CasOFFinder tool (http://www.rgenome.net/cas-offinder/).^69^ Briefly, 20-bp spacer sequences for the top sgRNA candidate without PAM sequences were used as the query using hg38 as the reference genome for canonical *Sp*Cas9 PAM sites. The algorithm was executed using 3 or fewer mismatches and DNA and RNA bulge sizes of 2. The list of off-target genes was then overlayed with all genes with hypermethylated promoters.

### Statistical analysis

Statistical analyses were performed in Prism 10 (GraphPad Software, San Diego, CA) and RStudio version 2023.12.1 + 402. Statistics are presented as the mean ± SD. Targeted assessments were performed in biological triplicates or sextuplets. Genome-wide assessments were performed in sextuplets. Between-group differences were analyzed using a one-way analysis of variance (ANOVA). Statistical differences between the means of the two groups were determined using an independent samples t test. The *p* value cut-off for all targeted analyses was set at *p* < 0.05. Statistical analyses of differentially methylated sites were performed using the methylKit with a Fisher exact test using a Benjamini-Hochberg correction. Individual CpGs were considered differentially methylated when *q* value <0.01 and %methylation difference >25%. Statistical analyses of differentially expressed genes were performed using DESeq2 in RStudio 3.6.0. The null hypothesis was rejected for tests with FDR <1%.

## Data Availability

To review GEO accession GSE283225: https://www.ncbi.nlm.nih.gov/geo/query/acc.cgi?acc=GSE283225. Enter token uhmdyguwbtmxjwz into the box.

## Acknowledgments

The authors would like to acknowledge their various funding sources NINDS 5R01NS102486–05, R24 201603716, CIRM Bridges Trainee Funding, A Stewart’s and Dake Family Gift, HELP4HD International, WeHaveAFace.org, The Dickenson’s Catalyst Fund, and philanthropic donors from the 10.13039/100004792HD community, including the Roberson family and Team KJ. Authors would also like to acknowledge Biorender (biorender.com) for graphic generation and all graphs were generated with Prism v.10.3.1.

## Author contributions

J.J.W. and K.D.F. conceived the project and designed the experimental strategy. J.J.W., J.A.N.M.H., and K.D.F. prepared the manuscript with contributions from A.S., C.E.G., Y.-A.C., S.A.C., and J.A.N. J.J.W. and J.A.N.M.H. analyzed expression data from multiple cell lines. J.J.W. and Y.-A.C. cloned plasmids for transfection experiments. J.J.W. and C.E.G. performed cell culture maintenance. J.J.W. performed transfection experiments and ChIP-qPCR experiments. J.J.W., A.S., Y.-A.C., and S.A.C. performed qPCRs for gene expression analysis. J.A.N.M.H. analyzed the RRBS data and assisted J.J.W. with data visualization.

## Declaration of interests

The authors declare no competing interests.

## Supplemental information

Table S1. Normalized read counts of reduced representative bisulfite sequencing of CRISPRoff treated 293s
